# Performance of Modified Early Warning Score (MEWS) and Circulation, Respiration, Abdomen, Motor, and Speech (CRAMS) score in trauma severity and in-hospital mortality prediction in multiple trauma patients: a comparison study

**DOI:** 10.7717/peerj.7227

**Published:** 2019-06-25

**Authors:** Xiaobin Jiang, Ping Jiang, Yuanshen Mao

**Affiliations:** 1 Emergency Department 1, Shanghai Ninth People’s Hospital, Shanghai JiaoTong University School of Medicine, Shanghai, China; 2 Research into Artifacts, Center for Engineering (RACE), The University of Tokyo, Kashiwa, Chiba, Japan; 3 Department of Urology, Shanghai Ninth People’s Hospital, Shanghai JiaoTong University School of Medicine, Shanghai, China

**Keywords:** Scoring system, MEWS, CRAMS, Multiple trauma

## Abstract

**Background:**

With an increasing number of motor vehicle crashes, there is an urgent need in emergency departments (EDs) to assess patients with multiple trauma quickly, easily, and reliably. Trauma severity can range from a minor to major threats to life or bodily function. In-hospital mortality and trauma severity prediction in such cases is crucial in the ED for the management of multiple trauma and improvement of the outcome of these patients. Previous studies have examined the performance of Modified Early Warning Score (MEWS) or Circulation, Respiration, Abdomen, Motor, and Speech (CRAMS) score based solely on mortality prediction or injury severity prediction. However, to the best of our knowledge, the performances of both scoring systems on in-hospital mortality and trauma severity prediction have not been compared previously. This retrospective study evaluated the value of MEWS and CRAMS score to predict in-hospital mortality and trauma severity in patients presenting to the ED with multiple traumatic injuries.

**Methods:**

All study subjects were multiple trauma patients. Medical data of 1,127 patients were analyzed between January 2014 and April 2018. The MEWS and CRAMS score were calculated, and logistic regression and receiver operating characteristic curve analysis were conducted to investigate their performances regarding in-hospital mortality and trauma severity prediction.

**Results:**

For in-hospital mortality prediction, the areas under the receiver operating characteristic curve (AUROCs) for MEWS and CRAMS score were 0.90 and 0.91, respectively, indicating that both of them were good in-hospital mortality predictors. Further, our study indicated that the CRAMS score performed better in trauma severity prediction, with an AUROC value of 0.84, which was higher than that of MEWS (AUROC = 0.77). For trauma severity prediction, the optimal cut-off value for MEWS was 2, while that of the CRAMS score was 8.

**Conclusions:**

We found that both MEWS and CRAMS score can be used as predictors for trauma severity and in-hospital mortality for multiple trauma patients, but that CRAMS score was superior to MEWS for trauma severity prediction. CRAMS score should be prioritized in the prediction of trauma severity due to its excellence as a multiple trauma triage tool and potential contribution to rapid emergency rescue decisions.

## Introduction

Multiple trauma is reported as the fifth leading cause of mortality in China (Wang, Pan & Pan, 2017; Yin, Liang & Liu, 2015). Over 400,000 people die from trauma caused by motor vehicle crashes or industrial accidents each year in China, among which multiple trauma patients comprise about 1.0–1.8% (Zhang, Hong & Gregory, 2017; Yingcheng et al., 2014). Emergency departments (EDs) in China are faced with challenges in the management of multiple trauma due to its high mortality risk.

An efficient trauma triage system aims to support medical personnel in the identification of life-threatening conditions, performing timely assessments and prioritization of treatment according to the severity of the patient’s medical condition (Wangara et al., 2019). Because multiple trauma is critical and complex, early and proper triage of multiple trauma patients must be carried out as soon as the patient is admitted to the ED.

However, there is still no standard trauma triage tool that can be promptly and easily used by emergency physicians, surgeons, and intensivists to improve the morbidity and mortality in EDs. Various scoring systems, mainly based on vital signs, anatomical score, and neurological score, have been developed and used as trauma triage tools. Anatomical-based scoring systems include the abbreviated injury scale (Gennarelli & Wodzin, 2006) and its derived score injury severity score (ISS) (Wang, Pan & Pan, 2017; Yin, Liang & Liu, 2015; Yingcheng et al., 2014). ISS is used for injury severity assessment (Yin, Liang & Liu, 2015; Yingcheng et al., 2014) and mortality prediction in elderly patients (Wang, Pan & Pan, 2017), but requires injury site diagnosis by trauma specialists which is inconvenient and time-consuming in the ED. Neurological-based scoring systems include Alert, Confused, Drowsy, Unresponsive (ACDU) scales; Alert, Confused, Pain and Unresponsive (AVPU) scales; and Glasgow Coma Scale (GCS) (Kelly, Upex & Bateman, 2004; McNarry & Goldhill, 2004, Raman et al., 2011). ACDU and AVPU are derived from the GCS, and GCS is superior to AVPU (Zadravecz et al., 2015) and favored for brain trauma triage in elderly patients (Wasserman et al., 2015). The GCS is useful for evaluating damage to the central nervous system and determining prognosis, but is susceptible to interference by ethanol, drugs, tracheal intubation, and other factors (GX, GT & ZM, 2015). In addition, sophisticated scoring systems (Jones, Trzeciak & Kline, 2009; Goodacre, Turner & Nicholl, 2006; Olsson, Terént & Lind, 2004; Olsson & Lind, 2003; Shapiro et al., 2003; Boyd, Tolson & Copes, 1987; Long, Bachulis & Hynes, 1986; Sartorius et al., 2010; Hung et al., 2017; Knaus et al., 1985) incorporating both vital signs and neurologic or anatomic-based scores, such as trauma score (TS) (Long, Bachulis & Hynes, 1986), trauma and injury severity score (Boyd, Tolson & Copes, 1987), acute physiology and chronic health evaluation (APACHE) II score (Knaus et al., 1985), Rapid Acute Physiology Score (RAPS) (Goodacre, Turner & Nicholl, 2006), Rapid Emergency Medicine Score (REMS) (Olsson & Lind, 2003), and Mortality in Emergency Department Sepsis Score (MEDS) (Shapiro et al., 2003), have been proposed for mortality prediction and injury severity assessment. TS is simple and fast to use but is easily affected by physiological compensation, hypovolemia, hypoxia, tracheal intubation, and other factors. MEDS is mainly used in ED patients with suspected sepsis, especially for suspected infection cohorts (Shapiro et al., 2003). REMS and RAPS are mainly used in non-surgical patients; REMS is derived from RAPS. Further, REMS is superior to RAPS for mortality prediction in emergency medical admissions (Goodacre, Turner & Nicholl, 2006) and in-hospital mortality prediction (Olsson, Terént & Lind, 2004), and MEDS is superior to both of them for in-hospital mortality prediction for splenic abscess patients (Hung et al., 2017). REMS has the same predictive accuracy as the APACHE II score for in-hospital mortality prediction (Olsson & Lind, 2003). These scoring systems have been reported as reliable tools for mortality prediction and injury severity assessment, but are complicated and inconvenient for calculation (e.g., APACHE II requires 14 variables), which make it difficult to meet the needs of a rapid risk stratification tool in a regular ED setting.

Faced with the requirement of a simple, rapid, and effective trauma triage tool in the ED, Modified Early Warning Score (MEWS) (Subbe et al., 2001) and Circulation, Respiration, Abdomen, Motor, and Speech (CRAMS) score (Gormican, 1982) have been widely used for mortality prediction and trauma severity assessment in China. MEWS is a modified version of the early warning score (Goldhill et al., 2005). MEWS has been used to assess hospital admission (Subbe et al., 2001; Burch, Tarr & Morroni, 2008), in-hospital mortality (Burch, Tarr & Morroni, 2008; Le Onn Ho et al., 2013), detecting pre-hospital critical illness (Fullerton et al., 2012), fast track care for femoral fracture patients (Ollivere et al., 2012), and severe conditions of patients in intensive care unit (Tavares et al., 2008). On the other hand, CRAMS score has been used to triage trauma patients (Clemmer et al., 1985), and both retrospective and prospective studies have shown that CRAMS score is a trauma triage tool which is easy to use and accurate in identifying major trauma victims with high specificity and sensitivity (Oprita, Aignatoaie & Gabor-Postole, 2014). Both MEWS and CRAMS score are meritorious in that they can be calculated immediately because each of the variables in these scoring systems can be measured simply and rapidly, allowing the quick clinical determination of critically-ill patients requiring urgent intervention (Fullerton et al., 2012; Subbe et al., 2001; Oprita, Aignatoaie & Gabor-Postole, 2014). However, previous literature has investigated the performance of MEWS or CRAMS score based solely on mortality prediction or trauma severity prediction. To the best of our knowledge, no study has compared the performance of both systems in predicting in-hospital mortality and injury severity. Further, the patients examined previously were mainly trauma patients rather than multiple trauma patients.

In this study, we evaluated the value of MEWS and CRAMS score for predicting in-hospital mortality and trauma severity in patients presenting to the ED with multiple traumatic injuries, and compare the performance of MEWS and CRAMS score for assessing trauma severity and predicting in-hospital mortality in the victims.

## Materials and Methods

### Ethical statement

This retrospective research project was approved by the research ethics committee of Shanghai Ninth People’s Hospital, affiliated to Shanghai JiaoTong University School of Medicine (approval no.: 2018146-T132). The need for informed consent from study participants was waived. All data were processed anonymously.

### Settings and subjects, and study design

Shanghai Ninth People’s Hospital is a non-profit university-affiliated tertiary teaching hospital located in Huangpu and Baoshan district in Shanghai, China. Its ED has 50 beds and is the second largest emergency center in Shanghai, equipped with medical detection devices and advanced emergency treatment instruments (www.9hospital.com.cn). The hospital admits approximately 20,000 trauma patients, 1.5% of which suffer from multiple trauma each year.

All adult medical patients consecutively admitted to the ED at Shanghai Ninth People’s Hospital, who had been conclusively diagnosed with multiple trauma from January 2014 to April 2018, were studied retrospectively to compare MEWS and CRAMS score outcomes for trauma injury severity and in-hospital mortality prediction. Here, imaging examinations, such as computed tomography (CT) scan, were used to diagnose multiple trauma by checking for the presence of two or more separate injuries, and if one or a combination of more than one endangered the patient’s life.

**Figure 1:**
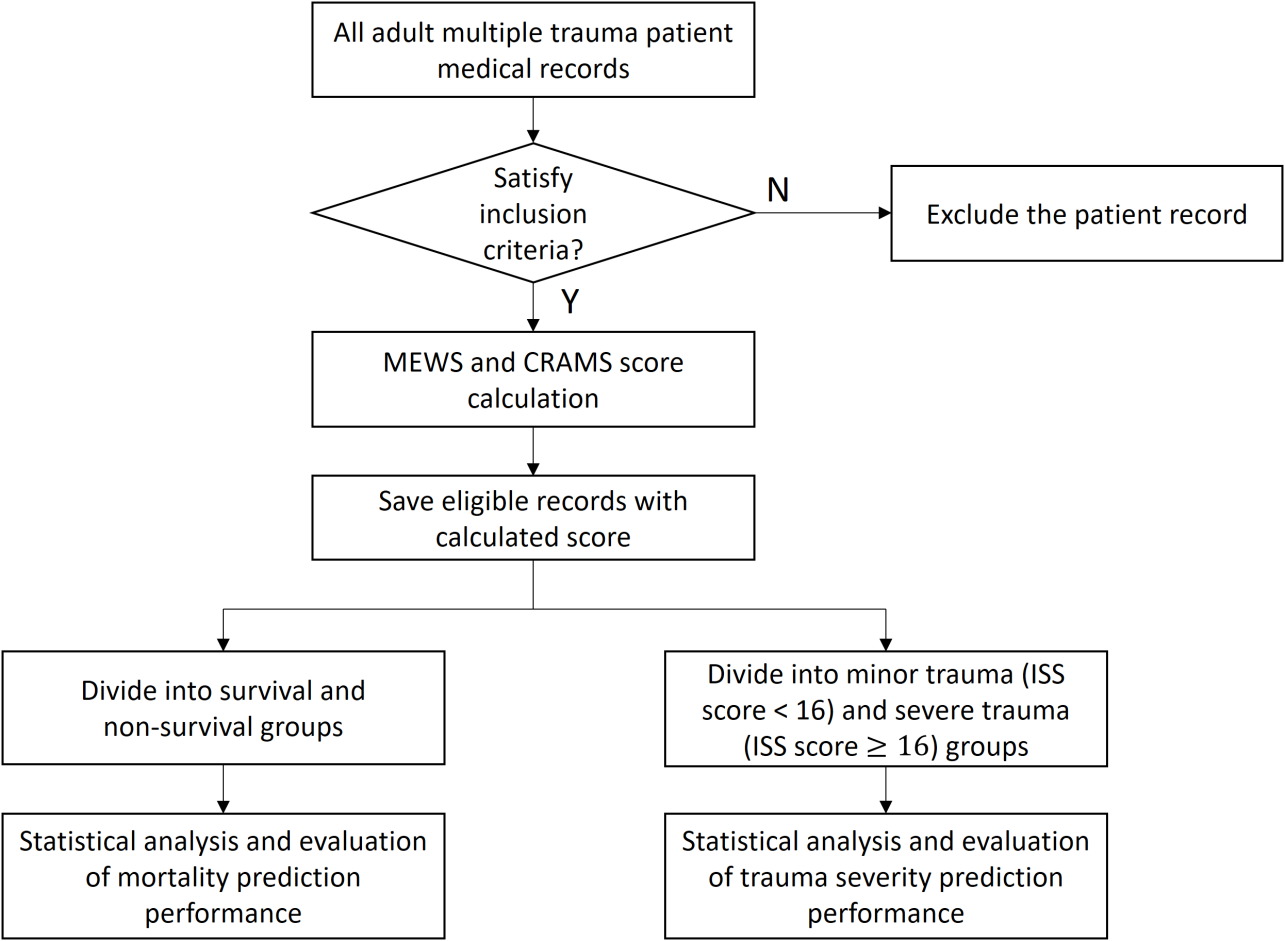
Study design.

Figure 1 shows the flow diagram of the study. Inclusion criteria were: clear history of trauma, and final diagnosis of multiple injuries examined by imaging; age not younger than 16 years; complete clinical and medical history; no stroke; no dysfunction of the heart, liver, kidney, or other important organs before injuries; and no sepsis, pneumonia, and other histories of systemic infections before injuries. This study focused on the outcomes of multiple trauma patients so that only patients finally diagnosed with multiple trauma were included. The heart rate (HR) and systolic blood pressure (SBP) in the MEWS and SBP in the CRAMS is based on a range of normal adult values, so only patients aged sixteen years or older were included. Missing data with missing values of all vital signs related to MEWS and CRAMS score were excluded because they could not be imputed. Further, patients with stroke; dysfunction of the heart, liver, kidney, or other important organs before injuries; and sepsis, pneumonia, and other history of systemic infections before injuries were excluded, because it was difficult to identify the cause-and-effect of the in-hospital mortality and trauma severity. Further, we aimed to investigate in-hospital mortality, so dead-on-arrival patient data were excluded.

The MEWS and CRAMS score were calculated for each subject based on the corresponding measured variables in their medical record (see sections on “MEWS” and “CRAMS score”). The medical records and calculated scores were saved to the database for further statistical analysis.

To investigate the outcome of in-hospital mortality prediction, the subjects were divided into survival and non-survival groups based on living condition within 28 days of hospitalization. Meanwhile, to investigate the outcome of trauma severity prediction, the same subjects were grouped into minor trauma and severe trauma groups based on the ISS—a “gold standard” among the anatomic injury severity indicators that is widely used in clinical science. The ISS was calculated using electronic medical records based on the diagnosis by imaging detection as well as surgical results. The ISS was only used for trauma classification in this study. Among the subjects, an ISS score < 16 was classified as minor trauma, while an ISS score ≥ 16 was classified as severe trauma (Baker et al., 1974; Gennarelli & Wodzin, 2006; Baker & O’Neill, 1976; Copes et al., 1988).

### MEWS

The MEWS is widely used in the clinical setting as a quantified scoring system based on HR (beats per minute), SBP (mmHg), respiratory rate (RR, cycles per minute), temperature (T, °C), and AVPU. As reported previously, the AVPU is estimated from the GCS as follows: *A* = 14–15, *V* = 9–13, *P* = 4–8, *U* = 3 (Kelly, Upex & Bateman, 2004; McNarry & Goldhill, 2004; Raman et al., 2011; Wasserman et al., 2015; Zadravecz et al., 2015). The corresponding score, ranging from zero to three, for each variable is shown in Table 1 (Subbe et al., 2001; Clemmer et al., 1985).

**Table 1:** Modified early warning score (MEWS).

Variable	Score			
	0	1	2	3
Systolic blood pressure (mmHg)	101–199	81–100	70–80	<70
			≥200	
Heart rate (/min)	51–100	40–50	<40	≥130
		101–110	111–129	
Respiratory rate (/min)	9–14	15–20	<9	≥30
			21–29	
Temperature (°C)	35–38.4		<35	
			≥38.5	
AVPU score	Alert	Reacts to voice	Reacts to pain	Unresponsive

### CRAMS score

The CRAMS score was calculated based on the following five variables: circulation, RR, abdomen, motor, and speech. Among these, circulation, RR, and speech are particularly important parameters for monitoring the health status of trauma patients (Gormican, 1982; Clemmer et al., 1985; Peng et al., 2017). The corresponding score, ranging from zero to two, for each variable is shown in Table 2. In contrast to MEWS, a lower CRAMS score indicates worse state of patients.

**Table 2:** Circulation, Respiration, Abdomen, Motor, and Speech (CRAMS) score.

Variable	Score		
	2	1	0
Circulation	Normal capillary refill and SBP ≥ 100	Delay capillary refill or 85 ≤ SBP < 100	No capillary refill or SBP < 85
Respiratory rate	Normal	Labored or shallow or >35 bpm	Absent
Abdomen	Abdomen and thorax nontender	Abdomen or thorax tender	Abdomen rigid or flail chest
Motor	Normal	Responds only to pain, no posturing	No response or postures
Speech	Normal	Confused or inappropriate	No or unintelligible sounds

### Statistical analysis

The performance of MEWS and CRAMS score for in-hospital mortality and trauma severity prediction among multiple trauma patients were compared. Categorical variables were compared using Pearson’s chi-squared test and were described as frequencies (%). Numerical variables were compared by the non-parametric Mann–Whitney’s *U*-test and reported as median (interquartile range (IQR)).

Modified Early Warning Score and CRAMS score for all eligible subjects were computed based on Tables 1 and 2 and were compared using the non-parametric Mann–Whitney’s *U*-test and were reported as median (IQR).

Missing data with missed values of all four vital signs (T, HR, RR, and SBP) were excluded because they could not be imputed. Multiple imputation was performed to handle the missing data with missed values containing less than four vital signs.

To investigate the predictive values of MEWS and CRAMS score for trauma severity, logistic regression analysis was performed and the areas under the receiver operating characteristic curve (AUROCs) were evaluated for minor and severe trauma. To investigate the predictive values of MEWS and CRAMS score for in-hospital mortality, the same logistic regression and AUROC analysis framework was used based on survival and non-survival. The following model was employed for logistic regression analysis:

(1) $$p = {1 \over {1 + {e^{ - ({\rm\beta _0} + {\rm\beta _1}{{\rm{X}}_1})}}}}$$

where β_0_ is the intercept; β_1_ is the score coefficient; and *X*_1_ is the score.

The R version 3.5.2 (www.r-project.org), a free software environment for statistical computing and graphics, was used to perform the logistic regression, and the R project package *pROC* (Robin et al., 2018) was used to compute AUROCs, and sensitivity, specificity, and accuracy rates for the corresponding optimal cut-off points.

## Results

### Setting and subjects

**Figure 2:**
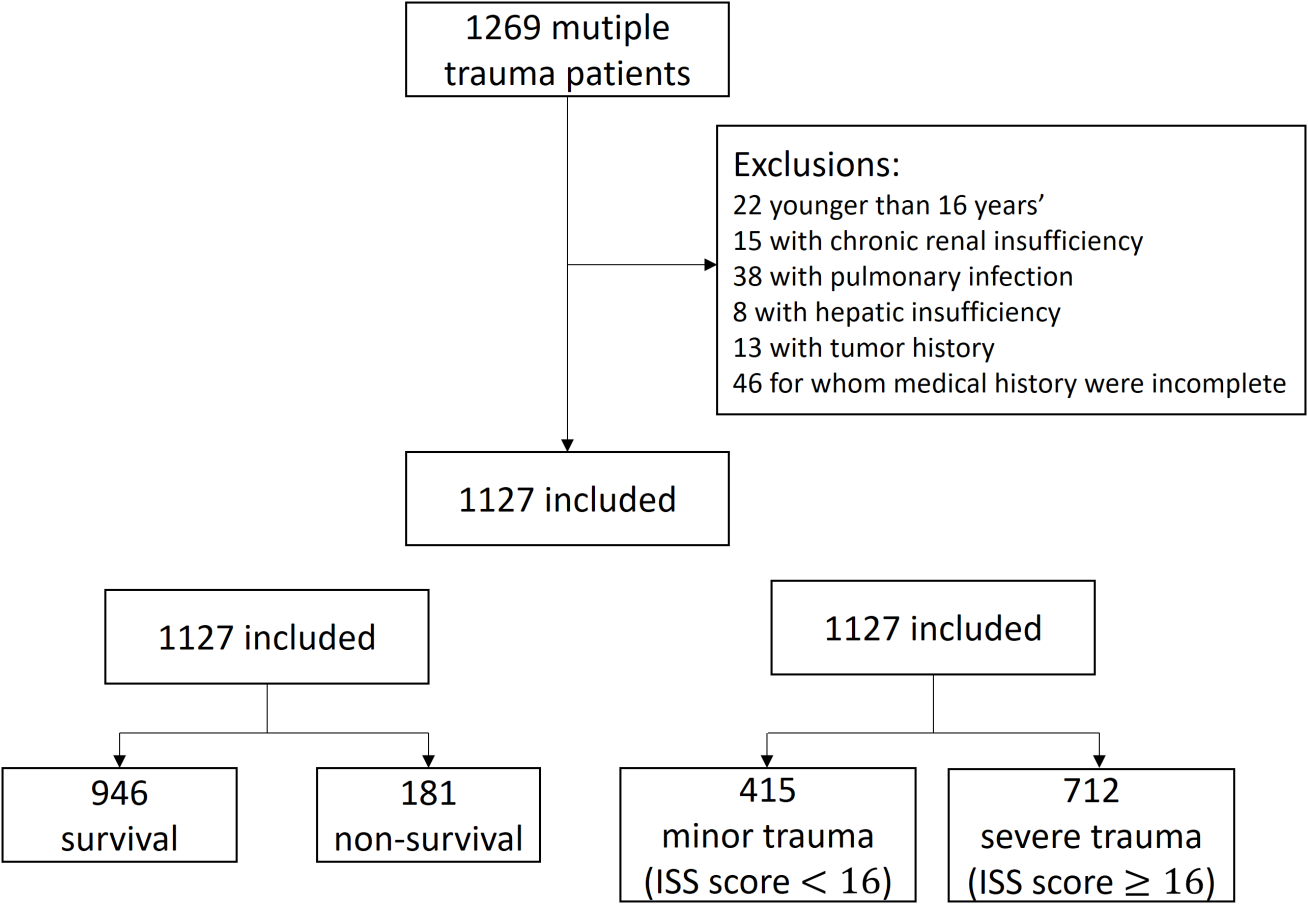
Study population.

Figure 2 describes our study population. Medical records of 1,269 patients with multiple trauma were collocated, and 1,127 cases satisfying the inclusion criteria were included. The study population comprised 73.7% males, with a median (IQR) age of 48 (38–59). All patients underwent ground transportation without air transportation. Most patients (91.5%) were transported by ambulance. The median (IQR) length of stay (LOS) of the patients was 12 (4–21) days. Patients in severe trauma group (LOS, 14.5 (3–25) days) were admitted for a longer duration than those in the minor trauma group (LOS, 9 (5–15) days). Majority of patients sustained injuries in motor vehicle crashes (55.5%). The primary injury sites of both survivors and non-survivors were the head and neck (39.7% vs. 70.2%, respectively), while those of minor trauma and severe trauma populations were bony pelvis and extremities (43.1%) and the head and neck (58.3%), respectively. There were 51 (4.5%) patients with missing data, including 30 with missing values of three vital signs, 10 with missing values of two vital signs, and 11 with missing values of one vital sign.

To investigate the outcome of in-hospital mortality prediction, the 1,127 cases were divided into survival and non-survival groups. The 1,127 eligible cases included 946 (83.9%) survivors and 181 (16.1%) non-survivors. Meanwhile, to investigate the outcome of trauma severity prediction, the same 1,127 cases were divided into minor trauma (ISS score < 16) (415 (36.8%) patients) and severe trauma (712 (63.2%)) groups.

Table 3 shows the characteristics of the study population in the survival and non-survival groups. Comparison of the survivors and non-survivors revealed the following factors to be statistically significant (*p* < 0.05) (Median (IQR))–RR: 20 (18–20) vs. 25 (20–29); temperature: 37.0 (36.8–37.0) vs. 36.5 (36.0–37.0) °C; and AVPU: 0 (0–0) vs. 3 (2–3).

**Table 3:** Characteristics of the survival and non-survival groups.

	Total	Survival	Non-survival	*P*-value
No.	1,127	946	181	
Male, *n* (%)	831 (73.7)	697 (73.7)	134 (74.0)	0.994
Age (years)	48 (38–59)	48 (38–58)	48 (37–62)	0.494
Temperature (°C)	36.9 (36.7–37.0)	37.0 (36.8–37.0)	36.5 (36.0–37.0)	<0.001
Heart rate (/min)	84 (75–96)	84 (75–94)	87.5 (68–117)	0.065
Respiratory rate (/min)	20 (18–20)	20 (18–20)	25 (20–29)	<0.001
Systolic blood pressure (mmHg)	130 (114–148)	130 (116–147)	124 (89–159)	0.099
AVPU	0 (0–1)	0 (0–0)	3 (2–3)	<0.001
LOS	12 (4–21)	14 (8–22)	1 (0–4)	<0.001
Time of transport (hour)	2 (1–3)	2 (1–3)	1 (1–1)	<0.001
Cause of injury, *n* (%)				0.001
Motor vehicle crashes	626 (55.5)	511 (54.0)	115 (63.5)	
High fall	213 (18.9)	183 (19.3)	30 (16.6)	
Crushing injury	32 (2.8)	20 (2.1)	12 (6.6)	
Cut/pierce	41 (3.6)	35 (3.7)	6 (3.3)	
Burn	3 (0.3)	2 (0.2)	1 (0.6)	
Tumble injury	149 (13.2)	141 (14.9)	8 (4.4)	
Struck	63 (5.6)	54 (5.7)	9 (5.0)	
Primary injury site, *n* (%)				<0.001
Face	18 (1.6)	18 (1.9)	0 (0.0)	
Head and neck	503 (44.6)	376 (39.7)	127 (70.2)	
Thorax	267 (23.7)	239 (25.3)	28 (15.5)	
Abdomen and visceral pelvis	106 (9.4)	93 (9.8)	13 (7.2)	
Bony pelvis and extremities	232 (20.6)	220 (23.3)	12 (6.6)	
External structures	1 (0.1)	0 (0.0)	1 (0.6)	
Discharge status, *n* (%)				<0.001
Expired in the hospital	181 (16.1)	0 (0.0)	181 (100.0)	
Discharge home	524 (46.5)	524 (55.4)	0 (0.0)	
Discharge against medical advice	67 (5.9)	67 (7.1)	0 (0.0)	
Discharge home with self-care	311 (27.6)	311 (32.9)	0 (0.0)	
Transfer to another hospital	44 (3.9)	44 (4.7)	0 (0.0)	
Score				
MEWS	2 (1–3)	1 (1–2)	5 (4–8)	<0.001
CRAMS	9 (7–10)	9 (8–10)	5 (4–6)	<0.001

**Note:**

Data are reported as median (IQR).

Table 4 shows the characteristics of the study population in minor trauma and severe trauma groups. Upon comparison of minor trauma and severe trauma, the following factors were found to be statistically significant (*p* < 0.05) (Median (IQR))–SBP: 136 (121–151) vs. 127 (107–145) mmHg; RR: 20 (19–20) vs. 20 (18–21); HR: 82(75–90) vs. 85 (75–100); temperature: 37.0 (36.8–37.0) vs. 36.8 (36.5–37.0) °C; and AVPU: 0 (0–0) vs. 0 (0–2).

**Table 4:** Characteristics of the minor trauma and severe trauma groups.

	Total	Minor trauma	Severe trauma	*P*-value
No.	1,127	415	712	
Male, *n* (%)	831 (73.7)	279 (67.2)	552 (77.5)	<0.001
Age (years)	48 (38–59)	48 (38–60)	48 (38–58)	0.496
Temperature (°C)	36.9 (36.7–37.0)	37.0 (36.8–37.0)	36.8 (36.5–37.0)	<0.001
Heart rate (/min)	84 (75–96)	82 (75–90)	85 (75–100)	0.019
Respiratory rate (/min)	20 (18–20)	20 (19–20)	20 (18–21)	0.003
Systolic blood pressure (mmHg)	130 (114–148)	136 (121–151)	127 (107–145)	<0.001
AVPU	0 (0–1)	0 (0–0)	0 (0–2)	<0.001
LOS	12 (4–21)	9 (5–15)	14.5 (3–25)	<0.001
Time of transport (hour)	2 (1–3)	2.0 (1.0–2.0)	2.0 (1.0–3.0)	0.005
Cause of injury, *n* (%)				<0.001
Motor vehicle crashes	626 (55.5)	187 (45.1)	439 (61.7)	
High fall	213 (18.9)	66 (15.9)	147 (20.6)	
Crushing injury	32 (2.8)	6 (1.4)	26 (3.7)	
Cut/pierce	41 (3.6)	27 (6.5)	14 (2.0)	
Burn	3 (0.3)	2 (0.5)	1 (0.1)	
Tumble injury	149 (13.2)	95 (22.9)	54 (7.6)	
Struck	63 (5.6)	32 (7.7)	31 (4.4)	
Primary injury site–*n* (%)				<0.001
Face	18 (1.6)	17 (4.1)	1 (0.1)	
Head and neck	503 (44.6)	88 (21.2)	415 (58.3)	
Thorax	267 (23.7)	88 (21.2)	179 (25.1)	
Abdomen and visceral pelvis	106 (9.4)	42 (10.1)	64 (9.0)	
Bony pelvis and extremities	232 (20.6)	179 (43.1)	53 (7.4)	
External structures	1 (0.1)	1 (0.2)	0 (0.0)	
Discharge status, *n* (%)				<0.001
Expired in the hospital	181 (16.1)	2 (0.5)	179 (25.1)	
Discharge home	524 (46.5)	261 (62.9)	263 (36.9)	
Discharge against medical advice	67 (5.9)	29 (7.0)	38 (5.3)	
Discharge home with self-care	311 (27.6)	95 (22.9)	216 (30.3)	
Transfer to another hospital	44 (3.9)	28 (6.7)	16 (2.2)	
Score				
MEWS	2 (1–3)	1 (1–2)	2 (1–4)	<0.001
CRAMS	9 (7–10)	10 (9–10)	8 (6–9)	<0.001

**Note:**

Data are reported as median (IQR).

### MEWS and CRAMS score

Table 3 and 4 show the MEWS and CRAMS score of the study population. Comparing survivor and non-survivor populations, the median MEWS (IQR) was 1 (1–2) vs. 5 (4–8) (*p* < 0.001); the median CRAMS score (IQR) was 9 (8–10) vs. 5 (4–6) (*p* < 0.001).

Comparing minor trauma with severe trauma, the median MEWS (IQR) score was 1 (1–2) vs. 2 (1–4), *p* < 0.001; the median CRAMS (IQR) was 10 (9–10) vs. 8 (6–9), *p* < 0.001.

**Figure 3:**
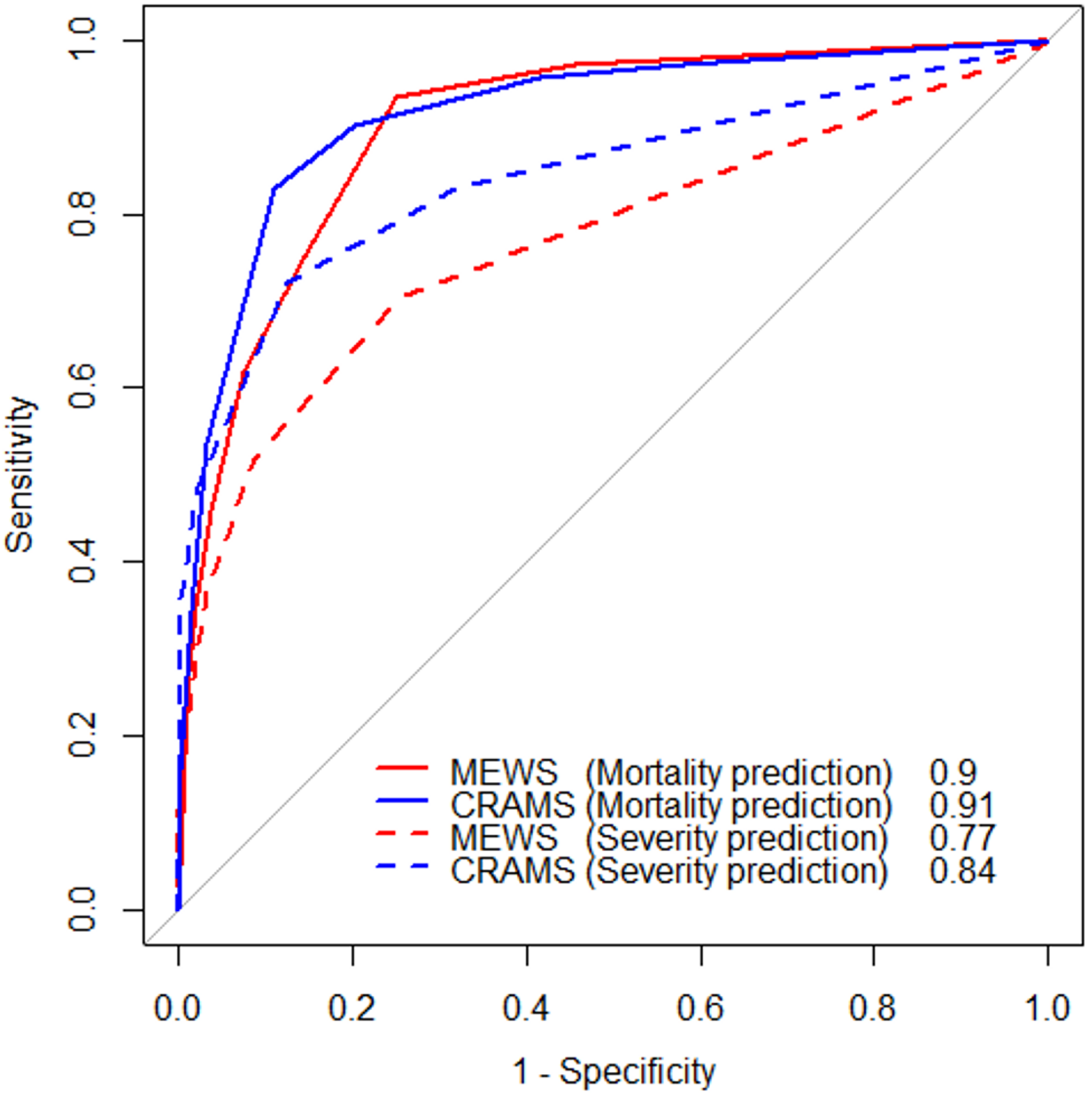
Receiver operating characteristic (ROC) curve of the study population. The red solid line is the ROC curve of MEWS in-hospital mortality prediction. The blue solid line is the ROC curve of CRAMS in-hospital mortality prediction. The red dashed line is the ROC curve of MEWS trauma severity prediction. The blue dashed line is the ROC curve of CRAMS trauma severity prediction.

Figure 3 shows the receiver operating characteristic curves of the study population. For in-hospital mortality prediction, the AUROCs of MEWS and CRAMS score were 0.90 (95% confidence interval [CI] [0.88–0.92]) and 0.91 (95% CI [0.89–0.94]), respectively, indicating that both MEWS and CRAMS score were good predictors of in-hospital mortality. For multiple trauma severity prediction, the AUROCs of MEWS and CRAMS score were 0.77 (95% CI [0.74–0.79]) and 0.84 (95% CI [0.82–0.87]), respectively, indicating that CRAMS score was a better predictor of trauma severity. Table 5 shows the optimal cut-off values and their corresponding accuracy, sensitivity, and specificity values. For in-hospital mortality prediction, the optimal cut-off values of MEWS and CRAMS score were ≥3 and ≤6, respectively. For multiple trauma severity prediction, optimal cut-off values of MEWS and CRAMS score were ≥2 and ≤8, respectively.

**Table 5:** Optimal cut-off values and their corresponding sensitivities, specificities, and accuracy rates for in-hospital mortality and trauma severity prediction.

	In-hospital mortality prediction				Trauma severity prediction			
	Optimal cut-off	Accuracy	Sensitivity	Specificity	Optimal cut-off	Accuracy	Sensitivity	Specificity
MEWS	≥3	0.78	0.93	0.75	≥2	0.72	0.70	0.74
CRAMS	≤6	0.88	0.83	0.89	≤8	0.78	0.72	0.87

## Discussion

The ED is the main location for treatment of emergency trauma patients, and trauma injury severity is an essential factor for evaluating the quality of trauma treatment and for predicting prognosis in the ED, especially for patients with multiple injuries (Boyd, Tolson & Copes, 1987; Long, Bachulis & Hynes, 1986).

In contrast to previous studies (Le Onn Ho et al., 2013; Ollivere et al., 2012; Sartorius et al., 2010; Steyerberg et al., 2008) that mainly focused on mortality prediction or injury severity prediction outcomes of the scoring systems, the present study, to the best of our knowledge, is the first to evaluate the performance of MEWS and CRAMS score in both in-hospital mortality prediction and trauma severity prediction. Further, our study included multiple trauma patients.

Our results demonstrated that both MEWS and CRAMS score were good predictors of in-hospital mortality, based on the AUROC values of 0.90 and 0.91, respectively. However, the trauma severity prediction performance of CRAMS score was better than that of MEWS. Furthermore, the performances of MEWS and CRAMS score in in-hospital mortality prediction were better than their performances in severity prediction. Hence, both MEWS and CRAMS score can be used for in-hospital mortality prediction, whereas CRAMS score can be used for trauma severity prediction with higher priority while triaging multiple trauma patients in the ED.

Both MEWS and CRAMS score are good predictors of in-hospital mortality because they both include vital signs (e.g., SBP and RR) and neurological variables (e.g., AVPU, Motor, and Speech), which are strongly related to mortality risk. Nevertheless, we argue that the CRAMS score is a better predictor of trauma severity due to the additional inclusion of the anatomical variable (i.e., Abdomen, a scoring criteria for abdominal and thoracic severity), which might enhance the injury assessment of CRAMS score. Further evaluation will be conducted to investigate this view in the future work.

Furthermore, CRAMS score is more convenient and rapid than MEWS because of fewer vital sign measurements and simpler score calculation. CRAMS score requires fewer vital sign measurements (2 (SPB and RR) vs. 4 (SBP, HR, RR, and T)) and fewer score stratifications for calculation (3 (0–2) vs. 4 (0–3)) than MEWS. Previous studies have demonstrated that CRAMS score can be assessed by the medical staff of the ED within 1–2 min of arrival of injured patients at the hospital, and it has high maneuverability because it is not limited by instruments and the location of injured patients (Burch, Tarr & Morroni, 2008; Tavares et al., 2008; Goldhill et al., 2005).

No standard multiple triage method is available in EDs in China. This study presented two rapid and convenient scoring systems for multiple trauma patient management. Our results showed that patients meeting the cut-off MEWS ≥ 3 or CRAMS score ≤ 6 should be treated as critically wounded patients; even if their vital signs are relatively stable, they might require immediate medical intervention, prompt imaging (e.g., whole-body CT), as well as preparation for surgery. Furthermore, among patients predicted to survive (CRAMS score > 6), those with CRAMS score ≤ 8 should be given more attention with respect to monitoring for disease-related changes for urgent rescue.

However, our study is not devoid of limitations. This retrospective study was confined to small samples. Although there was no difference in pre-hospital treatment, factors such as varying times of transition to the hospital may have resulted in bias of the analysis. In addition, the retrospective nature of the study is a limitation as it was not conducted in real time and results of scoring by multiple readers in a crunch could have been different. The population size is also a limiting factor because data were obtained from a single hospital. A larger sample from multiple hospitals is required to confirm our findings. We plan to collect more data for further investigation of injury severity prediction by MEWS and CRAMS score.

## Conclusions

Both MEWS and CRAMS score can be used as predictors of trauma severity and in-hospital mortality for multiple trauma patients. CRAMS score is superior to MEWS for trauma severity prediction and should be prioritized when predicting trauma severity. It can serve as an excellent multiple trauma triage tool and will contribute to rapid emergency rescue decisions.

## Supplemental Information

10.7717/peerj.7227/supp-1 Supplemental Information 1 Raw data of the study population. All variables related to MEWS and CRAMS calculation. Please refer to the CodeBook for the specific description of each variable (column label).

10.7717/peerj.7227/supp-2 Supplemental Information 2 The codebook to describe the usage of the raw data. The meaning of each column label is described in this codebook.

## Additional Information and Declarations

### Data Availability

The following information was supplied regarding data availability: All patient data are available in the Supplemental File.
