# Supplementary material for: Performance of Modified Early Warning Score (MEWS) and Circulation, Respiration, Abdomen, Motor, and Speech (CRAMS) score in trauma severity and in-hospital mortality prediction in multiple trauma patients: a comparison study
Source: PeerJ. 2019 Jun 25;7:e7227. doi: 10.7717/peerj.7227 (PMC6598668; doi:10.7717/peerj.7227)
Supplement: Supplemental Information 2 — The meaning of each column label is described in this codebook. [file peerj-07-7227-s002.docx]

Categorical variable

| Name | Value | Description |
| --- | --- | --- |
| Gender | 0 | Male |
|  | 1 | Female |
|  |  |  |
| Cause (Injury cause) | 0 | Traffic accident |
|  | 1 | High fall |
|  | 2 | Crushing injury |
|  | 3 | Cut/pierce |
|  | 4 | Burn |
|  | 5 | Tumble injury |
|  | 6 | Struck |
|  |  |  |
| InjurySite | 1 | Face |
|  | 2 | Head and neck |
|  | 3 | Throax |
|  | 4 | Abdomen and visceral pelvis |
|  | 5 | Bony pelvis and extremities |
|  | 6 | External structures |
|  |  |  |
| Mode of transport | 0 | By the police |
|  | 1 | By the ambulance car |
|  | 2 | By a private car |
|  | 3 | Transferred from other hospitals |
|  | 4 | By himself/herself |
|  |  |  |
| Discharge status | 0 | Expired in the hospital |
|  | 1 | Discharge home |
|  | 2 | Discharge against medical advice |
|  | 3 | Discharge home with self-care |
|  | 4 | Transfer to another hospital |
|  |  |  |
| Survival | 0 | Survival group |
|  | 1 | Non-survival group |
|  |  |  |
| Severity | 0 | Minor trauma group |
|  | 1 | Severe trauma group |

Numerical variable

| Name | Description |
| --- | --- |
| Age | Age (years) |
| T | Temperature ($℃$) |
| HR | Heart rate (/min) |
| RR | Respiratory rate (/min) |
| SBP | Systolic Blood Pressure (mmHg) |
| AVPU | AVPU score |
| Abdomen | Abdomen score in CRAMS |
| Move | Move score in CRAMS |
| Speech | Speech score in CRAMS |
| AIS | Abbreviated injury scale (9 sites) |
| ISS | Injury severity score (6 sites) |
| MEWS | Calculated MEWS score |
| CRAMS | Calculated CRAMS score |
| LOS | Length of stay (day) |
| ICU LOS | Length of stay in ICU (day) |
| Time of transport | Time of transport (hour) |
